# Excretion of glucose analogue with SGLT2 affinity predicts response effectiveness to sodium glucose transporter 2 inhibitors in patients with type 2 diabetes mellitus

**DOI:** 10.1007/s00259-023-06256-7

**Published:** 2023-05-17

**Authors:** Barbara Katharina Geist, Helmut Brath, Lucia Zisser, Josef Yu, Barbara Fueger, Lukas Nics, Eva Maria Patronas, Alexandra Kautzky-Willer, Marcus Hacker, Sazan Rasul

**Affiliations:** 1 Department of Biomedical Imaging and Image-Guided Therapy, Division of Nuclear Medicine, Medical University of Vienna, Waehringer Guertel 18-20, 1090 Vienna, Austria; 2 Diabetes & Metabolic Outpatient Clinic, Health Centre Vienna South, Vienna, Austria; 3 Department of Biomedical Imaging and Image-Guided Therapy, Division of General and Pediatric Radiology, Medical University of Vienna, Vienna, Austria; 4 Department of Internal Medicine III, Division of Endocrinology and Metabolism, Gender Medicine Unit, Medical University of Vienna, Vienna, Austria

**Keywords:** Diabetes type 2, PET, SGLT2 inhibitors, Sodium glucose cotransporter 2, Therapy response

## Abstract

**Purpose:**

Sodium-glucose cotransporter 2 inhibitor (SGLT2i) regulation, developed as treatment for patients with type 2 diabetes, can be imaged with the glucose analogue alpha-methyl-4-deoxy-4-[^18^F]fluoro-d-glucopyranoside (Me4FDG), a positron emission tomography (PET) tracer with a high affinity for SGLT1 and SGLT2 proteins. With regard to therapy effectiveness, we aimed to investigate whether clinical parameters or Me4FDG excretion could predict response to SGLT2i in patients with type 2 diabetes.

**Methods:**

In a longitudinal, prospective study, 19 patients with type 2 diabetes underwent Me4FDG combined PET and magnetic resonance imaging (PET/MRI) scans at baseline and 2 weeks after initiation of therapy with SGLT2i, accompanied by the collection of blood and urine samples. Me4FDG-excretion was determined from the Me4FDG uptake in the bladder. Long-term response was determined by HbA1c level after 3 months; a strong response to the therapy was defined as a reduction of HbA1c by at least 10% from baseline.

**Results:**

SGLT2i resulted in significantly increased Me4FDG excretion (4.8 vs. 45.0, *P* < 0.001) and urine glucose (56 vs. 2806 mg/dl, *P* < 0.001). Baseline urine glucose and baseline Me4FDG excretion correlated both with long-term decline in HbA1c with *r* = 0.55 (*P* < 0.05). However, only Me4FDG excretion was a predictor of a strong response to SGLT2i (*P* = 0.005, OR 1.9).

**Conclusions:**

Using Me4FDG-PET, we demonstrated for the first time renal SGLT2-related excretion before and after short-term SGLT2i treatment. In contrary to other clinical parameters, SGLT2-related excretion before treatment was a robust predictor of long-term HbA1c response in patients with type 2 diabetes, suggesting that therapy effectiveness is only dependent of endogenous SGLT2 processes.

**Supplementary Information:**

The online version contains supplementary material available at 10.1007/s00259-023-06256-7.

## Introduction

Treatments targeting inhibition of sodium-glucose cotransporter 2 receptors in renal tubules, commonly abbreviated as SGLT2i, have now been well studied and demonstrated remarkable effects on metabolic health as well as nephro- and cardioprotective effects in patients with and without type 2 diabetes mellitus.

Ordinarily, the consumed glucose, which enters the kidney via the blood stream, is first filtered in the renal glomeruli and completely reabsorbed from the renal tubule system. The resorption process is mainly performed by SGLT2 (90%) and to a lesser extent by SGLT1 (10%) [1, 2]. In pathological conditions such as type 2 diabetes, the glucose load in the blood exceeds the reabsorption capacity of the kidneys, leading to glucose being excreted in the urine and causing glucosuria. Inhibitors of SGLT2 obstruct these receptors in the renal tubule system, thereby rapidly reducing glucose reabsorption while increasing glucosuria. Consequently, as an immediate response of SGLT2i, there is a significant reduction in blood glucose levels and, interestingly, also an initial decrease in glomerular filtration rate (GFR), which is a reversible effect that is partially associated with hemodynamic effects of the therapy [3, 4]. In addition, several favorable long-term effects of SGLT2i such as weight loss and overall improvement in blood lipids, blood pressure, cardiac and renal function [5–8], and particularly the long-term reduction in HbA1c in most of the patients with type 2 diabetes receiving this therapy have been observed [9, 10]. In this respect, previous studies revealed diverse long-term response rate to this therapy with a decline of HbA1c levels by 0.60 to 1.20 depending on its baseline value [11].

Although the majority of reported side effects of SGLT2 inhibitors, mainly an enhanced susceptibility to fungal and bacterial infections of the genital tract, do not minor the necessity and value of SGLT2 inhibitors, more serious effects appeared in several studies: a potential increased risk of foot amputations was reported and there might be clinical implications in special risk groups like patients with neuropathic ulcerations, both highly debated effects that need further investigation [12]. However, if serious side effects become a crucial key consideration for the administration of SGLT2 inhibitors, an early identification of patients who might not benefit from a therapy is inevitable. Additionally, following the idea of “precision medicine,” it might be interesting to be able to predict the efficacy of a therapy before starting it.

In fact, the function of SGLT2 protein can be studied in vivo using positron emission tomography (PET) and the glucose analogue alpha-methyl-4-deoxy-4-[^18^F]fluoro-d-glucopyranoside (abbreviated as: Me4FDG). In contrary to the most commonly used glucose-analogue tracer 2-deoxy-2-[^18^F]fluoro-d-glucose (FDG), Me4FDG is a substrate with high affinity for SGLT1 and SGLT2, meaning that this tracer is specifically binding to SGLT proteins and thus the organ-specific Me4FDG uptake allows the evaluation of the according SGLT function [13]. Applying this method, SGLT2 reabsorption performance can be assessed indirectly from Me4FDG excretion by measuring the Me4FDG concentration in the urinary bladder. Thus, the more Me4FDG that enters the bladder, the less it is reabsorbed by SGLT2. To our knowledge, individual patient response to SGLT2i has never been studied using noninvasive PET imaging. With the help of FDG, short-term SGLT2i treatment has been shown to improve renal function, which was associated with a better response to this therapy, but no specific tracer or predictive parameter to determine this response has been investigated to date. Since it is currently unknown whether blood or excretion parameters at baseline or shortly after initiation of SGLT2i treatment might anticipate long-term response to this therapy, we conducted this prospective longitudinal study. We monitored kidney function as well as excretion parameters at baseline and 2 weeks after initiation of SGLT2i therapy and evaluated them regarding long-term response in patients with type 2 diabetes using Me4FDG-PET imaging.

## Materials and methods

### Patients

Twenty consecutive patients with type 2 diabetes were enrolled in this prospective clinical trial (EudraCT 2018–002,972-42, ethics number 1899/2018) and each patient provided written informed consent prior participating in the study. One patient dropped out of the study 1 week before the second scan. Therefore, complete imaging data were available for only 19 patients. Using a combined positron emission tomography/magnetic resonance imaging (PET/MRI) Biograph mMR (Siemens Healthcare Diagnostics GmbH, Germany), each patient underwent two whole-body PET scans with Me4FDG: one baseline scan before initiation of SGLT2i therapy and one follow-up scan exactly 2 weeks after the first imaging.

Inclusion criteria were adult patients with type 2 diabetes, HbA1c level > 6.2%, planned initiation with SGLT2i treatment, and intact renal function (serum creatinine < 1.5 mg/dl and urinary albumin-to-creatinine ratio < 300 mg/g in random urine sample). After recruitment, patient received their first PET scan within 1–2 weeks. Exclusion criteria were anatomically altered or damaged kidneys, corticosteroids, and diuretic therapies at the time of the study, MRI-unsafe implants such as pacemakers and implantable cardioverter-defibrillators, previously intolerant MRI contrast agents, claustrophobia, and pregnancy.

The clinical indication for initiating a new therapy with a SGLT2i was based only on metabolic needs, independent of the study goals, and was realized by experienced diabetologists in addition to existing other antidiabetic treatment, based on the current national and international (ADA/EASD) guidelines [14]. Collectively, 16 out of the 19 patients were treated with daily 10 mg of dapagliflozin and 3 patients received daily 10 mg of empagliflozin.

### Study design

**Fig. 1:**
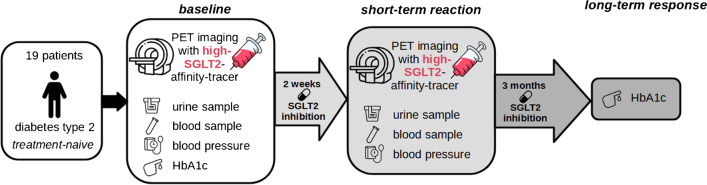
Study design. Short-term reaction to SGLT2i therapy was assessed 2 weeks after the baseline measurements with several blood values (including blood glucose), urine glucose, and blood pressure as well as with parameters of Me4FDG excretion derived from a PET/MRI scan. Long-term response was assessed with a HbA1c measurement three months after SGLT2i therapy start. SGLT2i, sodium-glucose cotransporter 2 inhibitors. PET/MRI, combined positron emission tomography/magnetic resonance imaging scan

As depicted in Fig. 1, patients acquired a baseline Me4FDG PET/MRI scan immediately before starting SGLT2i therapy. In addition, a fasting blood sample for measuring among others the baseline HbA1c as well as urine samples were collected. Blood pressure was measured prior to each scan. Me4FDG excretion was determined from the PET/MRI scans and GFR was evaluated from serum creatinine using the CKD-EPI formula.

To assess short-term responses to therapy, a second PET scan was performed using the same imaging protocol for Me4FDG excretion 2 weeks after initiation of therapy with SGLT2i. This was again accompanied by blood and urine samples and blood pressure measurements.

For estimation of long-term response, HbA1c was assessed 3 months after initiation of SGLT2i therapy. Response to SGLT2i therapy was defined as follows: a strong response if HbA1c decreased by equal or more than 10%, a moderate response as HbA1c declined less than 10%, and no response if HbA1c showed no change or an increase from baseline.

### Me4FDG radiosynthesis

Me-4-[^18^F]FDG was prepared in-house following a standardized protocol, using a GE FASTlab synthesizer (GE Healthcare, Boston, MA, USA) with dedicated disposable cassettes on the day of PET imaging. A regular FDG cassette was used and prepared as follows: The FDG-precursor-vial was removed and exchanged to a 11-mL crimp-vial, filled with a solution of 10 mg precursor for Me4FDG (β-d-galactopyranoside, methyl, 2,3,6-triacetate 4-trifluoromethanesulfonate, obtained from ABX advanced biochemical compounds GmbH, Radeberg, Germany) in 2 mL acetonitrile. Full radiopharmaceutical quality control according to the monographs of the European Pharmacopoeia (Ph. Eur.) was thoroughly performed prior to the release of the tracer and application to the patient. The production is not GMP compliant anymore but not necessary for in-house production within the General Hospital of Vienna.

### Me4FDG PET/MRI scan

After an intravenous hydration with 500 ml 0.9% NaCl (normal saline), participants were asked to empty their bladder directly before injection of 3 MBq/kg body weight of Me4FDG. A static PET acquisition for 2 min was performed 30 min after the injection of the glucose analogue tracer Me4FDG, accompanied by an MRI sequence for generation of an attenuation correction map. PET data were then reconstructed into a 172 × 172 × 127 matrix using the ordinary Poisson ordered subset expectation maximization (OP-OSEM) 3D algorithm (3 iterations, 21 subsets, Gaussian filter). Scatter and attenuation corrections were performed with Dixon-based MRI-attenuation correction. For image analysis, volumes of interest (VOIs) were manually drawn in the MRI images using the Hermes Hybrid Viewer (Hermes Medical Solutions AB, Stockholm, Sweden): total left and right kidney including the pelvis, total bladder, total liver, and total heart as well as a cubic VOI of 1 cm^3^ in the center of the vena cava superior. VOIs were copied to the PET images, from which the corresponding mean Me4FDG concentration in each delineated organ was measured, expressed as mean standardized uptake value (SUV). The mean SUV in the bladder was taken as measure for SGLT-related glucose excretion.

### Statistical analysis

All data were anonymized. Image analysis was performed by nuclear medicine and radiology specialist; data evaluation was performed by a medical physicist. For all statistical evaluations, IBM SPSS Statistics version 27.0 was used. Kolmogorov Smirnov tests were performed with all baseline and follow-up parameters to assess their distribution. Pearson’s correlation coefficient was conducted to study correlations between the measured parameters. A paired Student’s *t*-test was used to compare blood and urine measured parameters before and after starting the therapy with SGLT2i, independent sample *t*-test was used to compare differences between groups. Furthermore, logistic regression analysis was performed to find the predictor of response to therapy. In all analyses, a *P*-value < 0.05 was considered statistically significant.

## Results

**Fig. 2:**
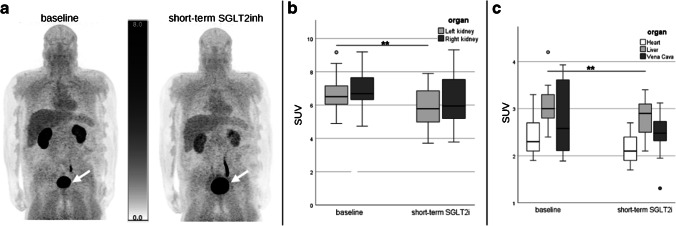
Me4FDG SUVs. **a** Total body PET images using Me4FDG of a patient with type 2 diabetes mellitus at baseline before therapy start (left) and after 2 weeks of therapy with SGLT2i (right). The white arrows mark the bladder, showing an uptake increase of Me4FDG after therapy start. Images are normalized to injected dose and body weight. **b** Boxplot of the Me4FDG SUV of the kidneys (left: light gray; right: dark gray), and of **c** total heart (white), total liver (gray) and the blood pool from vena cava (dark gray), before and after 2 weeks of SGLT2i. Highly significant differences with *P*-values ≤ 0.001 are marked with two asterisk on the according bars. PET, positron emission tomography; SGLT2i, inhibitors of sodium-glucose cotransporter 2. SUV, standardized uptake value

Nineteen patients (9 males, 10 females) with type 2 diabetes were included, all of them accomplished baseline and follow-up scans. Twelve patients reported a history of arterial hypertension, and 15 participants suffered from hypercholesterolemia, which had no impact on response or any clinical or PET parameter. In addition, 8 patients were former smokers and 2 subjects had chronic kidney disease. Additional clinical information on the patients studied is provided in the supplemental table (Table S1). In the PET scans, Me4FDG uptake was clearly visible in the kidneys, the bladder, and the liver; no uptake was found in the brain, poor uptake in the heart, and very low uptakes in the other depicted organs (Fig. 2). From all patients, baseline and follow-up blood samples as well as urine samples were available. Patient demographics are summarized in Table 1. No differences were found between male and female patients.

**Table 1:** Patient demographics as well as laboratory and PET parameters at baseline and 2 weeks after therapy initiation with SGLT2i. The according range is presented in parentheses

Parameters		Baseline	Short-term follow-up	*P*-value
Patient demographics	Age [years] Female: Male [*n*]	60 ± 14 (27–78) 10:9		
	BMI [kg/cm^2^]	32 ± 6 (25–44)	31 ± 6 (24–43)	0.3
Blood pressure	Systolic [mmHg]	150 ± 22 (114–186)	144 ± 21 (115–190)	0.2
	Diastolic [mmHg]	86 ± 10 (71–97)	85 ± 12 (64–112)	0.7
Blood values	Glucose [mg/dl]	151 ± 32 (88–221)	133 ± 34 (91–202)	0.03*
	ALP [U/l]	76 ± 26 (34–142)	73 ± 22 (52–128)	0.02*
	GGT [U/l]	39 ± 27 (13–86)	33 ± 22 (12–92)	0.01*
	Uric acid [mg/dl]	4.8 ± 1.4 (2.8–7.3)	4.2 ± 1.2 (2.0–6.6)	< 0.001*
	GFR [ml/min/1.73cm^2^]	97 ± 27 (54–152)	92 ± 27 (48–146)	0.06
Urine chemistry	Glucose [mg/dl]	56 ± 169 (3–730)	2806 ± 1216 (735–4836)	< 0.001*
	Albumin-to-creatinine [mg/g]	133 ± 420 (5–1824)	153 ± 415 (5–1643)	0.9
SUV	Liver	3.1 ± 0.4 (2.4–4.2)	2.8 ± 0.4 (2.1–3.4)	0.002*
	Heart	2.4 ± 0.4 (1.9–3.3)	2.1 ± 0.3 (1.7–2.7)	0.06
	Bladder (Me4FDG excretion)	4.8 ± 3.0 (1.7–10.8)	45.0 ± 22.5 (7.9–84.7)	< 0.001*
	Kidney left	6.7 ± 1.1 (4.9–9.2)	5.8 ± 1.2 (3.7–7.9)	0.004*
	Kidney right	7.0 ± 1.2 (4.7–9.2)	6.3 ± 1.5 (3.8–9.3)	0.08
	Blood pool (vena cava)	2.8 ± 0.7 (1.9–3.9)	2.5 ± 0.5 (1.3–3.1)	0.2

*BMI* body mass index, *GGT* gamma-glutamyltransferase, *ALP* alkaline phosphatase, *GFR* glomerular filtration rate, *PET* positron emission tomography, *SUV* standardized uptake value

^*^Significant values with a *P* < 0.05

### Short-term reaction to SGLT2i

Significant short-term reactions to SGLT2i therapy were found in several blood values and urine glucose; the values of blood glucose were significantly reduced, and the values of urine glucose were clearly increased 2 weeks after starting therapy with SGLT2i, see Table 1. However, values of blood pressure and BMI did not change significantly.

Regarding parameters obtained from the PET/MRI scans, SGLT2i led to a significant increase of Me4FDG excretion in terms of increased SUV value of urinary bladder (Fig. 2a) and to a significant reduction in SUV values of the liver and the left kidney, while the blood pool and the heart were not affected (Table 1, Fig. 2b, c). The SUV value of the right kidney was reduced, but not significantly (Table 1). Me4FDG excretion did not correlate significantly with blood glucose at baseline (*r* =  − 0.3, *P* = 0.08), but strongly correlated with the values of urine glucose (*r* = 0.92, *P* < 0.001). In general, blood glucose had only weak correlations with all measured values (*r* < 0.4).

### Baseline Me4FDG excretion predicted long-term response

Long-term response to SGLT2i based on values of HbA1c measured 3 months after the therapy initiation was available in all patients. From all baseline parameters collected before therapy start, only baseline Me4FDG excretion in terms of SUV value of the urinary bladder as well as baseline urine glucose levels correlated indirectly and significantly with HbA1c decline (*r* =  − 0.56, *P* = 0.018) and (*r* =  − 0.55, *P* = 0.027), respectively.

**Fig. 3:**
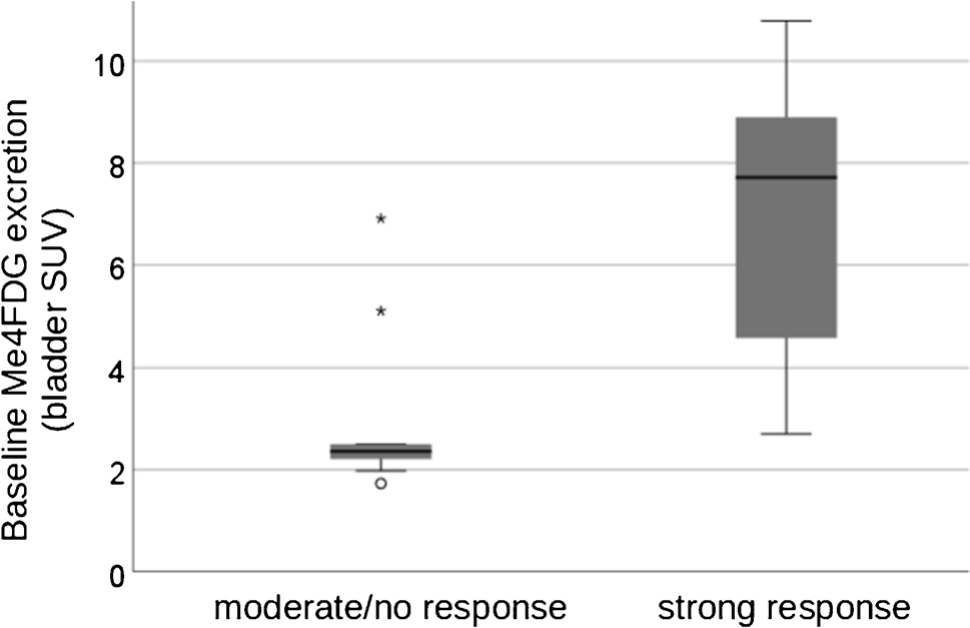
Boxplot of baseline Me4FDG excretion in patients with strong response (right) and moderate or no response (left). SGLT2i, inhibitors of sodium-glucose cotransporter 2. SUV, standardized uptake value

Collectively, 7 out of 19 studied patients exhibited a strong response to SGLT2i with a decline in HbA1c of more than 10%. Eight patients had a moderate response with a reduction of HbA1c value of up to 10%, and three patients had no response to SGLT2i therapy, i.e., no change or increase in HbA1c value. When baseline values of all patients who responded strongly to SGLT2i were compared with those who responded moderately or not to SGLT2i, a significant difference was found only for Me4FDG excretion (Table 2 and Fig. 3), whereas baseline values for blood glucose, HbA1c, or urine glucose showed no differences, and GFR, although higher in patients with strong response, failed to reach the significance level.

**Table 2:** Baseline parameters of 7 patients with strong response to SGLT2i and HbA1c reduction of equal and greater than 10% compared to the rest 11 patients

Parameters	Strong response (*n*: 7)	Moderate to no response (*n*: 11)	*P*-value
HbA1c [%]	9.0 ± 1.4	8.0 ± 1.1	0.11
Uric acid [mg/dl]	4.4 ± 1.5	4.9 ± 1.1	0.4
GFR [ml/min/1.73cm^2^] (mean ± SD)	113 ± 20	90 ± 25	0.059
Blood glucose [mg/dl] (mean ± SD)	153 ± 34	152 ± 32	0.9
Urine glucose [mg/dl] (mean ± SD)	111 ± 272	24 ± 56	0.3
Albumin-to-creatinine [mg/g]	45 ± 50	205 ± 570	0.5
Left kidney SUV (mean ± SD)	6.8 ± 1.0	6.6 ± 1.1	0.7
Right kidney SUV (mean ± SD)	7.1 ± 1.3	6.7 ± 1.2	0.6
Bladder SUV^$^(mean ± SD)	6.9 ± 3.0	3.0 ± 1.8	0.007*

*SD* standard deviation, *GFR* glomerular filtration rate, *SUV* standardized uptake value

^*^Significant values with a *P* < 0.05

^$^Urinary Me4FDG excretion

Patients who showed a strong decline in HbA1c of equal and more than 10% had a significantly higher Me4FDG excretion (measured with bladder SUV) before SGLT2i therapy compared to the rest of the patients. Based on the dichotomization of all patients into a group with strong response and a group including the remaining patients, a logistic regression model was performed, using those parameters, which showed significant or remarkable differences between both groups (i.e., baseline Me4FDG excretion and baseline GFR), and additionally albumin-to-creatinine. This showed that only the baseline Me4FDG excretion was a robust predictor for a strong response to SGLT2i, while other possible predictors (GFR, urine glucose, and albumin-to-creatinine) were not significant; for details, see Table 3.

**Table 3:** Results of the logistic regression models on strong response to SGLT2 inhibition using the parameters baseline Me4FDG excretion, baseline GFR, baseline urine glucose, and baseline albumin-to-creatinine

Parameter	Overall model significance	Odds ratios	95% confidence interval	*P*-value
Baseline Me4FDG excretion	0.005*	1.9	1.05–3.3	0.03*
Baseline GFR	0.046*	1.05	0.99–1.1	0.08
Baseline urine glucose	0.3	1.0	0.99–1.01	0.4
Baseline albumin-to-creatinine	0.4	1.0	0.99–1.00	0.6

^*^Significant values with a *P* < 0.05

*GFR* glomerular filtration rate

### Short-term reaction had no influence on long-term response

The long-term response to SGLT2i in terms of HbA1c decline after 3 months did not correlate with the changes of parameters from baseline to short-term follow-up, having correlation coefficients of *r* < 0.2 and *P* > 0.3. Only the change in uric acid of − 13% (from 4.8 mg/dl at baseline to 4.2 mg/dl at short-term follow-up) correlated with long-term response with *r* =  − 0.46, which failed the significance level with a *P*-value of *P* = 0.056.

## Discussion

We conducted this prospective, longitudinal study to assess the immediate impact of SGLT2i therapy on the function of organs such as kidney and liver and to find a predictor for long-term response to this therapy in patients with type 2 diabetes using blood and urine laboratory measurements as well as Me4FDG-PET scans.

Regarding short-term responses, as previously reported by others [3, 4], we observed a decrease in GFR values during SGLT2i therapy, but this was not statistically significant (*P* = 0.06, Table 1), possibly due to the fact that we reexamined patients after only 2 weeks of treatment. In addition, we observed an overall improvement of several blood parameters closely related to the patients’ general health, such as uric acid and gamma-GT, as previously described by other authors and in our own previous publication [15, 16]. Blood glucose levels decreased moderately, and urine blood glucose increased from 56 to 2806 mg/dl on average. Moreover, we could show that changes neither in levels of GFR nor in blood glucose are suitable parameters to predict long-term response to the therapy. This indicates that the long-term response is not linked to, or predicted by, the short-term response to SGLT2i. We could conclude that the intensity of a subject’s short-term reaction (i.e., within 2 weeks) to SGLT2i had no influence on the long-term response, 3 months after initiation of the therapy.

We also found that the short-term reduction in uric acid level, which was even lower but not significant in the patients with a strong response to therapy than in the other patients, correlated but only weakly (*r* =  − 0.46, *P* = 0.056) with the long-term HbA1c response. This may suggest that this immediate reduction in uric acid is likely resulting in a stronger long-term response. This effect was independent of baseline uric acid levels prior to initiation of therapy and requires to be further investigated over a longer duration. Moreover, we could not find any differences in the albumin-to-creatinine ratio, neither in a short-term response, nor between different responders. Since this parameter is a predictor for nephroprotection [17], we conclude that a strong response is also not dependent on the nephroprotective impact of SGLT2 inhibitors.

As expected, the Me4FDG excretion in terms of bladder SUV was also increased and correlated positively with the urine glucose. Interestingly, the SUV value of the left kidney significantly decreased 2 weeks after the starting therapy with SGLT2i that might reflect the reduction in the SGLT2-related reabsorption in the kidneys after this therapy. Note that the right kidney also showed a decline, but not significantly, which might be related to spill-over effects from the liver. In addition, two baseline parameters related to excretion like baseline Me4FDG excretion and baseline urine glucose correlated with long-term response. However, a logistic regression model assessing the prediction of a strong long-term response was significant only for Me4FDG excretion. Therefore, laboratory parameters reflecting only glucose (blood and urine glucose) levels cannot be considered robust predictors, whereas Me4FDG excretion was a proper predictor, a parameter mirroring pure SGLT2-related processes. Indeed, available evidence suggested individual differences in the expression of SGLT2 receptors and genetic variability of the SGLT2 polymorphism [18, 19]. This might lead to an uneven reabsorption of glucose among patients receiving SGLT2i therapy, which could consequently affect patients’ response to treatment and their perception of the drug’s side effects. As a result, despite the numerous reported beneficial long-term effects of SGLT2i on weight, blood pressure, and cardiac and renal functions, the individual experience with the benefits and adverse effects of this treatment varies widely. Although they are highly debated, there is some evidence that severe side effects could occur [12] in patients who received SGLT2i therapies. Hence, Me4FDG excretion might be a useful marker that directly mirrors the status of SGLT2 expression in patients with type 2 diabetes. In fact, body glucose load appears to be less prognostic than the genuine endogenous baseline renal SGLT2 activity, a value that can be readily ascertained with the tracer Me4FDG either from PET scans, or probably also via its radioactivity from urine samples, a hypothesis which certainly has to be addressed in further studies.

Since a high Me4FDG excretion might indicate low renal SGLT2-related reabsorption, this result suggests that a low baseline renal reabsorption capacity leads to a more effective long-term response of SGLT2i, which is independent of the short-term reactions or the baseline kidney function or the individual glucose levels. Essentially, chronic type 2 diabetes leads to an up to 20% increased renal reabsorption capacity [20]; thus, the longer a patient suffers from type 2 diabetes, the more glucose is reabsorbed via SGLT2 and the less is excreted from a failing SGLT2 function. Thus, we concluded that SGLT2i therapy is less effective when patients have long-standing type 2 diabetes or when their renal reabsorption capacity is overwhelmed. However, it might be difficult to correlate therapy effectiveness with disease duration, since it might not always be known how long a patient indeed was suffering from type 2 diabetes before diagnosis. However, to our knowledge, the effectiveness of SGLT2i therapy with regard to diabetes duration or severity was not studied yet.

Although this prospective longitudinal study was the first to use Me4FDG to measure levels of SGLT2 expression in patients with type 2 diabetes taking SGLT2i treatments, the study has several limitations that should be highlighted: First, the sample size of recruited patients is small (19 patients). Second, the study did not include a comparison arm with other antidiabetic therapies. Moreover, the effects of SGLT2i treatment on the function of other organs, which were not the focus of this study, might be displayed more clearly by Me4FDG PET scans. Third, we did not measure the radioactivity of the collected urine samples to investigate whether this parameter, as surrogate for the PET imaged bladder, could reproduce our results. Further clinical studies are therefore warranted, with larger sample size, considering other concomitant antidiabetic therapies of patients.

In conclusion, we measured renal SGLT2-related excretion at baseline and after short-term SGLT2i treatment in patients with type 2 diabetes using an SGLT2-specific radiotracer and PET scans for the first time. In contrast to blood and urine glucose levels, our results showed that only an assessment of pure endogenous SGLT2-related effects, in particular a high SGLT2-related excretion from the Me4FDG tracer before initiation of SGLT2i therapy, was a robust predictor of a strong long-term response to this antidiabetic treatment.

## Supplementary Information

Below is the link to the electronic supplementary material.

Supplementary file1 (DOCX 20 KB)

## Data Availability

The datasets generated during and/or analyzed during the current study are available from the corresponding author on reasonable request.
