# Supplementary material for: Excretion of glucose analogue with SGLT2 affinity predicts response effectiveness to sodium glucose transporter 2 inhibitors in patients with type 2 diabetes mellitus
Source: Eur J Nucl Med Mol Imaging. 2023 May 17;50(10):3034–41. doi: 10.1007/s00259-023-06256-7 (PMC10382381; doi:10.1007/s00259-023-06256-7)
Supplement: Supplementary file 1 — Supplementary file1 (DOCX 20 KB) [file 259_2023_6256_MOESM1_ESM.docx]

**Supplemental**

**Table S1:** Clinical characteristics of study participants at the time of the first Me4FDG PET scan before initiation of SGLT2i therapy

| **Patients** | **Gender** | **Age**  **years** | **Weight**  **Kg** | **BMI** | **HbA1c**  **%** | **Glucose mg/dl** | **Relevant**  **Comorbidities** | **Smoking Status** | **RAAS Therapies** |
| --- | --- | --- | --- | --- | --- | --- | --- | --- | --- |
| 1 | M | 77 | 80 | 36 | 7.2 | 137 | HTN, HCL | former | no |
| 2 | M | 62 | 130 | 38 | 10.4 | 152 | HTN, HCL, CHD | active | yes |
| 3 | F | 27 | 112 | 43 | 7.3 | 140 | HCL | never | no |
| 4 | F | 56 | 75 | 27 | 10.3 | 164 |  | active | no |
| 5 | M | 69 | 85 | 30 | 8.1 | 176 | HTN | active | yes |
| 6 | M | 74 | 78 | 25 | 7.6 | 116 | HTN, HCL | former | no |
| 7 | F | 54 | 78 | 27 | 8.1 | 120 | HCL | active | no |
| 8 | M | 73 | 80 | 27 | 7.1 | 151 | HTN, HCL | never | yes |
| 9 | F | 78 | 64 | 27 | 7.8 | 118 | HTN, HCL | never | yes |
| 10 | F | 64 | 86 | 28 | 8.9 | 196 | HCL | active | no |
| 11 | F | 53 | 96 | 32 | 8.4 | 166 | HTN, HCL | former | yes |
| 12 | M | 55 | 118 | 37 | 11.4 | 221 | HTN, HCL | former | yes |
| 13 | F | 67 | 60 | 25 | 7.6 | 121 | HTN, HCL | never | yes |
| 14 | F | 41 | 89 | 33 | 9.2 | 147 | - | active | no |
| 15 | F | 35 | 115 | 42 | 8.6 | 154 | - | never | no |
| 16 | F | 65 | 96 | 34 | 7.5 | 120 | HTN, HCL, CKD (G2) | former | no |
| 17 | M | 55 | 82 | 26 | 6.7 | 114 | HCL | former | yes |
| 18 | M | 77 | 103 | 33 | 7.6 | 214 | HTN, HCL, CKD (G2) | former | yes |
| 19 | M | 65 | 91 | 30 | 8.3 | 145 | HTN, HCL. | former | yes |

Kg: Kilogram; BMI: Body mass index; RAAS: Renin-angiotensin-aldosterone system inhibitors; M: Male; F: Female; HTN: Hypertension; HCL: Hypercholesterolemia; CHD: Coronary heart disease; CKD: Chronic kidney disease.
